# Formation of *Pseudomonas aeruginosa* Biofilms in Full-thickness Scald Burn Wounds in Rats

**DOI:** 10.1038/s41598-019-50003-8

**Published:** 2019-09-20

**Authors:** Kenneth S. Brandenburg, Alan J. Weaver, S. L. Rajasekhar Karna, Tao You, Ping Chen, Shaina Van Stryk, Liwu Qian, Uzziel Pineda, Johnathan J. Abercrombie, Kai P. Leung

**Affiliations:** Dental and Craniofacial Trauma Research and Tissue Regeneration Department, United States Army Institute of Surgical Research, 3650 Chambers Pass, Bldg 3610, JBSA Fort Sam, Houston, Texas 78234 USA

**Keywords:** Applied microbiology, Biofilms

## Abstract

Using Sprague-Dawley rats (350–450 g; n = 61) and the recently updated Walker-Mason rat scald burn model, we demonstrated that *Pseudomonas aeruginosa* readily formed biofilms within full-thickness burn wounds. Following the burn, wounds were surface-inoculated with *P. aeruginosa* in phosphate-buffered saline (PBS), while sterile PBS was used for controls. On post-burn days 1, 3, 7, and 11, animals were euthanized and samples collected for quantitative bacteriology, bacterial gene expression, complete blood cell counts, histology, and myeloperoxidase activity. Robust biofilm infections developed in the full-thickness burn wounds inoculated with 1 × 10^4^ CFU of *P. aeruginosa*. Both histology and scanning electron microscopy showed the pathogen throughout the histologic cross-sections of burned skin. Quantigene analysis revealed significant upregulation of alginate and pellicle biofilm matrix genes of *P. aeruginosa* within the burn eschar. Additionally, expression of *P. aeruginosa* proteases and siderophores increased significantly in the burn wound environment. Interestingly, the host’s neutrophil response to the pathogen was not elevated in either the eschar or circulating blood when compared to the control burn. This new full-thickness burn biofilm infection model will be used to test new anti-biofilm therapies that may be deployed with soldiers in combat for immediate use at the site of burn injury on the battlefield.

## Introduction

One of the greatest challenges facing the burn clinic is the complication of bacterial infection within the burn wound that may lead to more severe disease states, including sepsis. Scarcity of new antimicrobials, specifically anti-biofilm agents, further escalates the challenge posed by resistant microorganisms in treating burn wound infections. Several different animal models that seek to recapitulate the hallmarks of clinical burn wound infection are used to assist the development of novel antimicrobial therapies. The three most common burn wound infection *in vivo* models include pigs, mice, and rats^1–5^. Porcine models are often the best substitute for human skin, but their use for screening vast librarys of antimicrobials are hampered by high costs and ethical implications^3^. Mice offer the greatest range of benefits for characterizing aspects of the host’s response to burn infection, based on genetic mutants, but are often limited to acute (less than 48 hours post inoculation) burn wound infection before rapid onset of sepsis and death, potentially due to the requirement for injection of the high bacterial inoculums beneath the burn eschar to induce the burn infection^6–8^. Others have also included formulation of the pathogen in a biopolymer matrix to drive the infection towards a more chronic biofilm-like state^9,10^. However, these inoculation and exposure routes do not recapitulate what occurs in burn clinics and fail to mimic the natural development of infection in full-thickness burn injuries. Rats have played a significant role in the development and transition of antimicrobial burn wound treatments to clinics since the 1960’s, when the original Walker-Mason rat scald burn and surface infection model was described^11–16^.

The Walker-Mason rat scald burn model displays the classical features of invasive burn wound infection, typically observed with *Pseudomonas aeruginosa* using a natural surface route of infection, albeit with high inoculum levels of the pathogen, after induction of the burn injury. Importantly, studies using this model directly led to the development and clinical adoption of the ‘gold standard’ antimicrobial treatments for severe burn wounds, namely Sulfamylon® (mafenide acetate) and Silvadene® (silver sulfadiazine)^12,17–19^. These treatments greatly reduced the overall lethality of septic burn wound infection by *P. aeruginosa* in clinics^20^. While these antimicrobial therapies are proven effective in preventing infection, the immense number of daily person-hours and ease of application limits their utility outside a tier IV or V military treatment facility, including regional referral hospitals and hospitals within the United States^18,21^. Coupled with the difficulty in treatment deployability to forward operating theatres, any delay in application of the antimicrobial therapy to the burn injury may result in its decreased effectiveness, potentially due to the uninhibited development of bacterial biofilms within the burn eschar^12^.

We recently updated the Walker-Mason rat scald burn model in regards to skin preparation, pain relief, resuscitation regimen, and scald times for both deep partial- and full-thickness burn wound formation^22^. Our previous work showed that, within three days post-burn and surface inoculation with as little as 1 × 10^3^ CFU, the opportunistic pathogen *P. aeruginosa*, when left unimpeded, forms biofilms within deep partial-thickness burn wounds. Development of biofilm within the eschar was characterized by significant upregulation of genes encoding alginate (*alg8* and *algE*) and the iron binding siderophore pyoverdine (*pvdS*)^23–29^. These molecules contribute to the structure of the biofilm matrix as well as liberates bound iron from host tissues, respectively^25,26^. In this report, we expand our findings to include the infection kinetics and inoculum required of *P. aeruginosa* and its biofilm formation in full-thickness burn wounds. Similar to partial-thickness burn wounds, *P. aeruginosa* developed robust infections within the burn eschar, but the host’s neutrophil response to the pathogen seemed reduced compared to the partial-thickness burn infection, reported previously. Compared to partial-thickness burns, the invasiveness of the pathogen, leading toward sepsis, seemed heightened in the full-thickness burn injury by recovery of the pathogen in the blood in as little as 3 days post burn and inoculation most likely caused by greater burn wound tissue necrosis. Importantly, several key genes related to biofilm formation by *P. aeruginosa* were significantly upregulated in the tissue indicating development of mature biofilm in the full-thickness burn wound tissue.

## Results

**Figure 1:**
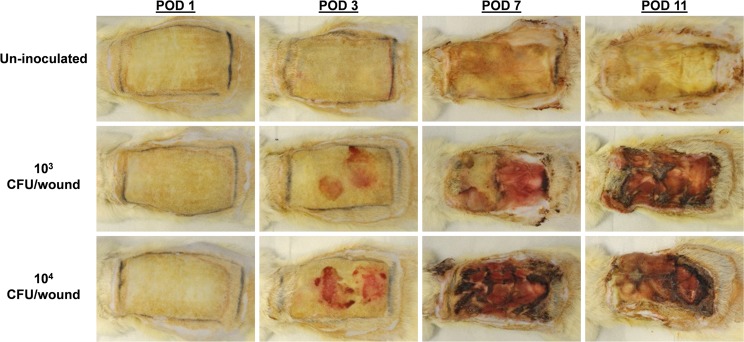
Representative gross images of full-thickness burn wounds inoculated with *P. aeruginosa* (1 × 10^3^ or 1 × 10^4^ CFU/wound) show significantly more wound exudate and areas of necrosis compared to wounds receiving sterile PBS (Un-inoculated).

We characterized the kinetics of *P. aeruginosa* infection and biofilm formation in full-thickness scald burns using the modified Walker-Mason burn model in Sprague-Dawley rats. Figure 1 shows representative gross appearance of the burn wound with and without *P. aeruginosa* infection over the course of the study. Exudate became apparent by POD 3 in the wounds inoculated with *P. aeruginosa*. By POD 7 and 11, areas of tissue necrosis within the burn wound could be easily observed in both inoculum groups compared to the controls. The un-inoculated wounds did show contamination of the eschar based on the discoloration of the burned area. Given the severity of the wound infection, an expected decline in body weight was observed in both inoculum groups, but generally leveled off or began to recover during the study with the extra administrations of pain medication and Lactated Ringers on POD’s 3 and 7. The body weights of the animals over the course of the infection are shown in Supplementary Fig. 1.

### Quantitative bacteriology

**Figure 2:**
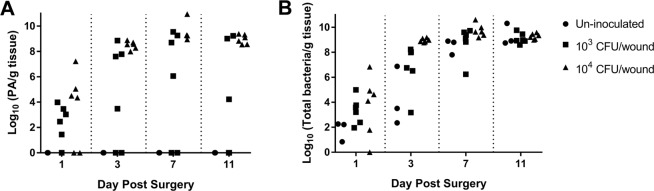
Maximum bacterial load of full-thickness burn wounds was achieved within 7 days. The viable counts from *P. aeruginosa* isolation agar (**A**) and non-selective agar (**B**) increase with time following the burn. *P. aeruginosa* was not cultured from un-inoculated control wounds. The 1 × 10^3^ inoculum showed pronounced variability in the *P. aeruginosa* counts due to lack of recovery during the second experimental run. The 1 × 10^4^ inoculum exhibited superior reproducibility, in terms of bacterial recovery, between all three experimental runs. Regardless of inoculation amount, by POD 11, all groups on the non-selective agar medium obtained a plateau of ~1 × 10^9^ CFU/g wound tissue of either *P. aeruginosa* or native flora.

**Figure 3:**
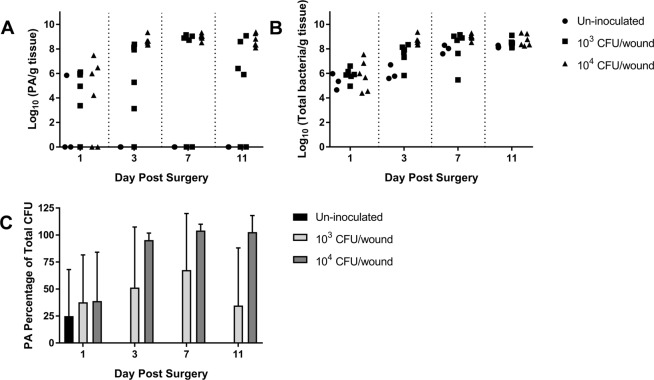
qPCR confirmed the *P. aeruginosa* and total bacterial loads within the burn eschar over 11 days following burn injury. The results of the qPCR analysis mirrored the data obtained in the viable counts for *P. aeruginosa* (**A**) and total bacterial cells (**B**). As a percentage of the total bacterial load (**C**), within three days post-burn and infection *P. aeruginosa* dominated the burn wounds inoculated with 1 × 10^4^ CFU/wound. As expected, wounds inoculated with 1 × 10^3^ CFU/wound showed greater variability of *P. aeruginosa* percentage of bacterial load due to its lack of recovery in the second experimental run.

Viable CFU counts isolated from the tissue biopsies are shown in Fig. 2. A similar increase in bacterial number was obtained on *P. aeruginosa* isolation agar (Fig. 2A) and standard blood agar (Fig. 2B) as seen previously in partial-thickness burn wounds infected with *P. aeruginosa*^22^. By POD 7, a plateau in number of microorganisms, *P. aeruginosa* or native flora, per gram of wound tissue was observed. Large variability in the 1 × 10^3^ CFU/wound inoculum was present in the counts obtained from *P. aeruginosa* isolation agar due to lack of *P. aeruginosa* recovery in the second run of the infection study. The results of the viable counts were confirmed via qPCR for bacterial DNA isolated from the homogenate of the same biopsy punches, shown in Fig. 3A,B. PCR analysis showed near perfect mirroring of the viable CFU counts. As a result of the variability within the 1 × 10^3^ inoculum, the *P. aeruginosa* percentage of total bacterial DNA recovered from the wound sample was greatly reduced compared to the 1 × 10^4^ inoculum, shown in Fig. 3C.

Table 1 documents the results of the Signal^TM^ Blood Culture System from the second and third runs of the study. Unfortunately, the Blood Culture System was not employed during the first experimental run, therefore blood culture results are not available from those animals. No animal showed positive results on POD 1. One of four animals in both inoculum groups showed positive blood cultures for *P. aeruginosa* on POD 3. By POD 7, 25% of 1 × 10^3^ CFU inoculum group was positive for *P. aeruginosa* in the blood stream, while 50% of the 1 × 10^4^ CFU inoculum group showed the pathogen in the blood. By POD 11, half of the animals in the 1 × 10^3^ CFU inoculum group showed *P. aeruginosa* and *Staphylococcus sciui ss sciuri* growth in the blood, while all animals in the 1 × 10^4^ CFU inoculum group were positive for *P. aeruginosa* or *Staphylococcus xylosus* in the blood. These data demonstrate that *P. aeruginosa* burn wound infection may lead to bacteremia by *P. aeruginosa* or other native flora^30,31^ [Sanjar *et al*. “Identification of Metagenomics Structure and Function Associated with Temporal Changes in Rat (Rattus norvegicus) Skin Microbiome during Health and Cutaneous Burn” *in press*] common to the rat skin that transgress to the blood stream within a week of the burn, if left untreated. Importantly, animals that received a burn but were not inoculated with *P. aeruginosa* did not show the same invasive burn wound infection and presence of any bacterial cells, *P. aeruginosa* or native microflora, in the blood stream.

**Table 1:** Results of Signal^TM^ Blood Culture System.

Group		POD 1	POD 3	POD 7	POD 11
Un-inoculated	Counts	0/2	0/2	0/2	0/2
	ID	—	—	—	—
10^3^ CFU/wound	Counts	0/4	1/4	1/4	2/4
	ID	—	*P. aeruginosa*	*P. aeruginosa*	*P. aeruginosa* *S. sciui ss sciuri*
10^4^ CFU/wound	Counts	0/4	1/4	2/4	4/4
	ID	—	*P. aeruginosa*	*P. aeruginosa*(2)	*P. aeruginosa*(3) *S. xylosus*

### Histology

**Figure 4:**
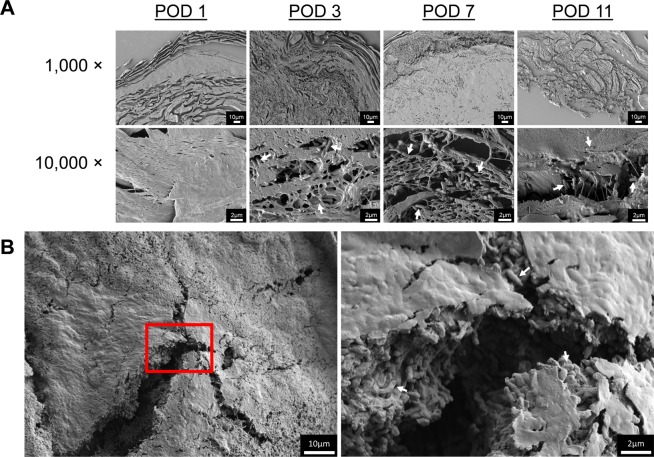
Scanning electron microscopy revealed the penetration of *P. aeruginosa* deep into the burn eschar via histologic cross-sections (**A**) of burn wounds inoculated with 1 × 10^4^ CFU/wound as well as dense biofilm matrix on the burn surface on POD 11 of the same inoculum group (**B**). In the tissue cross-sections, *P. aeruginosa* (arrows) invaded completely through the dermis and were associated with collagen fibers. Abundant numbers of *P. aeruginosa* (arrows) located on the surface of the burn were enmeshed in a smooth matrix material, possibly derived from both bacterial and host polysaccharides. Scale bar of 1,000× images is 10 µm and 10,000× images is 2 µm.

We evaluated histologic cross-sections of the burn wound inoculated with *P. aeruginosa* by scanning electron microscopy (SEM). Figure 4A shows both 1,000× and 10,000× magnification views obtained by SEM of a wound inoculated with 1 × 10^4^ CFU of *P. aeruginosa* over the course of the study. On POD 1, few if any bacterial cells were observed, but large numbers were seen penetrating into the eschar by POD 3. Similar to our work in partial-thickness burns, the bacterial cells were often associated with the collagen fibers of the burned tissue. We also examined the appearance of the eschar surface with SEM on POD 11. Figure 4B shows areas of both exposed bacterial cells and smoothed matrix like material that may be a combination of host proteins/tissues and bacterial exopolysaccharides of a wound inoculated with 1 × 10^4^ CFU of *P. aeruginosa*. Additionally, upon high magnification (10,000×), the layers of bacterial cells could be easily seen in the cracks on the surface of the eschar, potentially a product of the sample dehydration during the preparation for SEM imaging. Unlike the wounds infected with *P. aeruginosa*, SEM images of control burn wounds inoculated with PBS, Supplementary Fig. 2, do not show the presence of bacterial cells associated with the collagen fibers or within a matrix on the wound surface.

**Figure 5:**
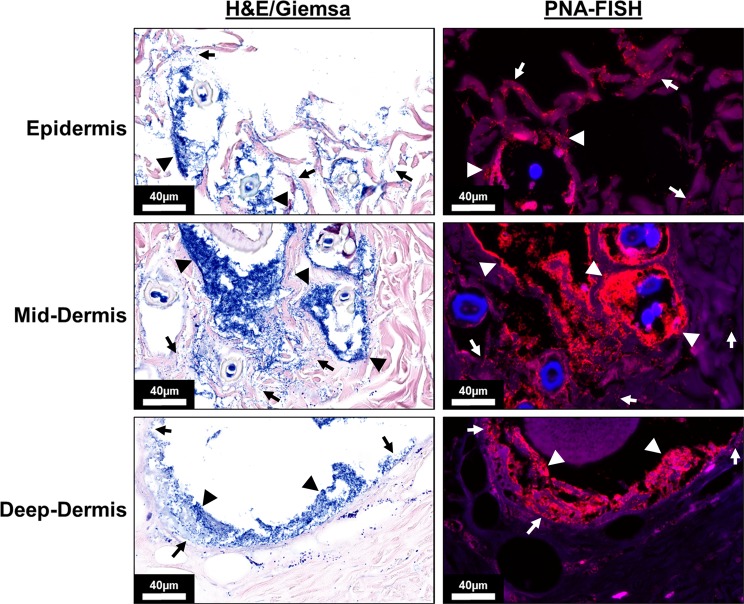
Representative micrographs of burn wound tissue cross-sections inoculated with 1 × 10^4^ CFU of *P. aeruginosa* cells reveal penetration of individual *P. aeruginosa* cells (arrows, stained blue in H&E/Giemsa and red in PNA-FISH) at all levels (Epidermis, Mid-Dermis, and Deep-Dermis) of the burned tissue by day 7 post-burn and inoculation. Bacterial aggregates (arrowheads) were observed at the epidermis, middle of the dermis, and in the deep dermis near the panniculus carnosus. Scale bar is 40 µm.

Micrographs of histologic sections from 1 × 10^4^ inoculated wounds on POD 7 stained with H&E and Giemsa or PNA-FISH are shown in Fig. 5. Micrographs of histologic sections from control un-inoculated wounds are shown in Supplementary Fig. 3 and an overview of the tissue sections are displayed in Supplementary Fig. 4. High magnification images taken with the 63× objective of three areas in the tissue section include the epidermis, the middle of the dermis, and of the deep dermis near the panniculus carnosus. The combination of the brightfield stains allows for identification of the tissue structure while also highlighting the penetration of the pathogen throughout all levels of the burned eschar. PNA-FISH, specific for *P. aeruginosa*, confirmed the identity of the pathogen within the tissue sections. Respective micrographs of the un-inoculated wounds on POD 7 after the burn are shown in Supplementary Fig. 3. The un-inoculated wounds did show the presence of the native flora, mostly gram-positive cocci, within the histological sections stained with H&E and Giemsa, but lacked the presence of the *P. aeruginosa* and microcolonies observed in the inoculated wounds stained with PNA-FISH.

### Biofilm gene expression

**Figure 6:**
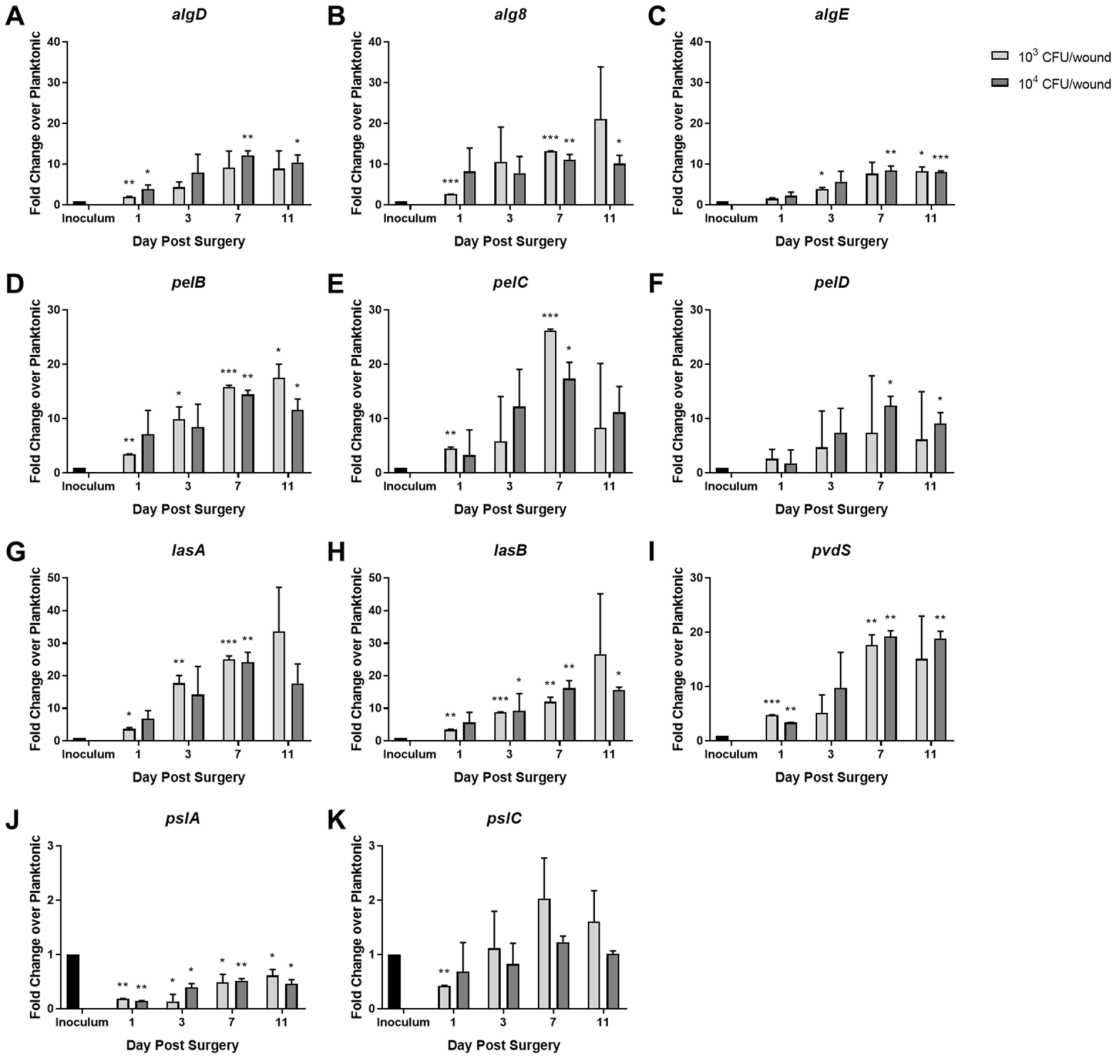
Increased expression of important *P. aeruginosa* biofilm genes detected in full-thickness burn tissue compared to the planktonic inoculum. Quantigene analysis showed increased expression of genes controlling production of alginate, *algD* (**A**), *alg8* (**B**), and *algE* (**C**); pel polysaccharide, *pelB* (**D**), *pelC* (**E**), and *pelD* (**F**); and virulence genes, *lasA* (**G**), *lasB* (**H**), and *pvdS* (**I**) in the full-thickness burn wound tissue compared to the planktonic inoculum. Interestingly, expression of psl polysaccharide genes *pslA* (**J**) and *pslB* (**K**) did not increase in the burn wound environment. (*p < 0.05; **p < 0.01, ***p < 0.001 in comparison with the planktonic inoculum).

Expression patterns of important biofilm associated genes of *P. aeruginosa* over the 11-day experiment are shown in Fig. 6 with results shown as a fold change of the planktonic inoculum. Alginate genes (*algD*, *alg8*, and *algE*) displayed in Fig. 6A–C show roughly 5–10 fold increases in expression levels as compared to the inoculum. Plateaus in *algD* and *algE* gene expression were reached by POD 7 after the burn. *Alg8* showed higher expression levels in the 1 × 10^3^ CFU/wound inoculated group as compared to the 1 × 10^4^ CFU/wound group, which were uniformly elevated ~10 fold higher than the inoculum throughout the experiment. Similar expression increases were observed in the *pelB*, *pelC*, and *pelD* (pellicle biofilm) genes over the course of the study as depicted in Fig. 6D–F. Roughly, 5–15 fold increases in pellicle biofilm gene expression were detected in the burn wound compared to the inoculum, although *pelC* and *pelD* showed increased variability in the 1 × 10^3^ inoculum. We measured the expression of three classical *P. aeruginosa* virulence genes (protease genes *lasA*, *lasB*, and iron starvation sigma factor gene *pvdS*) in the burn wound tissue, shown in Fig. 6G–I. Increased expression, between 10–30 fold over the inoculum, were measured over the course of the study for each virulence gene. Conversely, biofilm polysaccharide genes *pslA* and *pslC* did not show similar increases in expression level upon *in vivo* inoculation as seen in Fig. 6J,K, and may be reduced compared to the planktonic inoculum.

### Host neutrophil response

**Figure 7:**
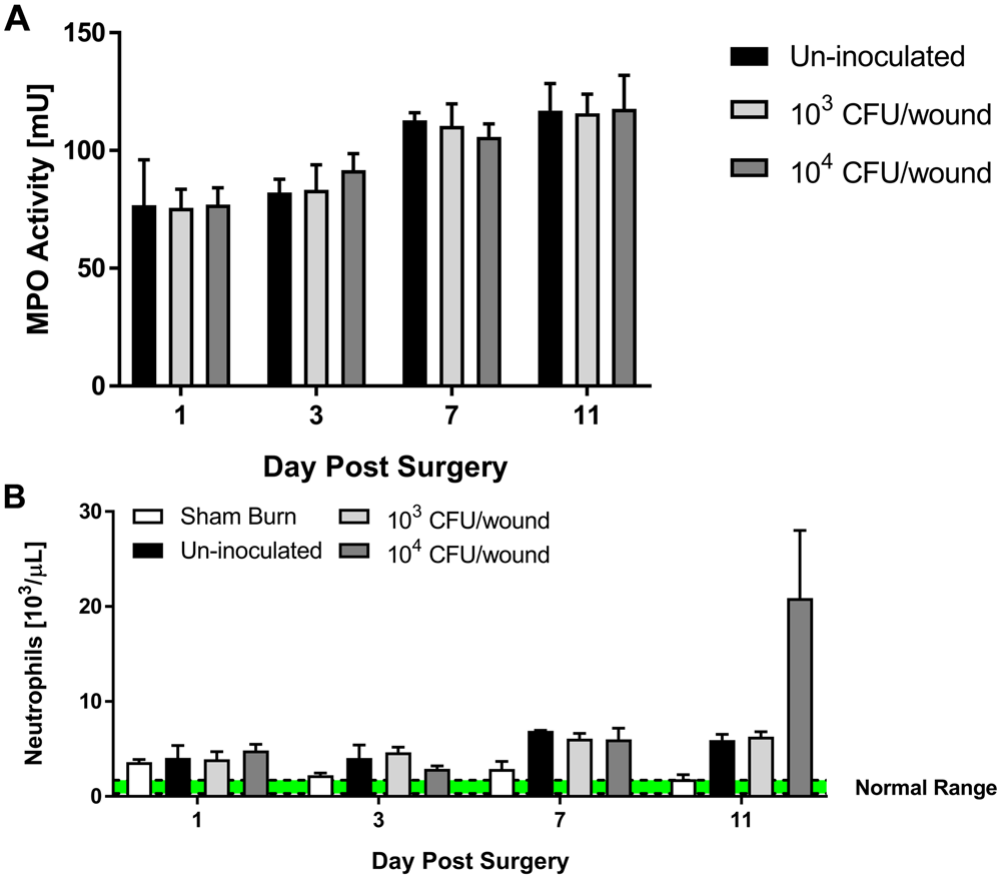
*P. aeruginosa* infection did not exacerbate the neutrophil response to full-thickness burn injury. General trends of increase MPO activity (**A**) were observed in full-thickness burn tissue, but were unaffected by concomitant *P. aeruginosa* infection. Slightly increased levels of circulating neutrophils (**B**) were measured following full-thickness burn injury but were not significantly increased with the presence *P. aeruginosa*.

The local tissue and systemic neutrophil response to the burn and *P. aeruginosa* infection are shown in Fig. 7. MPO activity confined to the burn wound, shown in Fig. 7A, was the same between all inoculation groups on each day. Generally, the MPO activity increased with time after the burn, but addition of *P. aeruginosa* did not further elevate Neutrophil MPO activity in the local tissue, regardless of inoculation level. Similar results were observed on a systemic level in the Neutrophil counts obtained from cardiac puncture as seen in Fig. 7B with only the 1 × 10^4^ CFU/wound inoculum showing increased circulating Neutrophils as compared to the un-inoculated burn wounds on POD 11.

## Discussion

We previously updated the Walker-Mason rat scald burn model to document the growth of *P. aeruginosa* biofilms in deep partial-thickness burn wounds^22^. This current study builds on that foundation, extending our *P. aeruginosa* infected animal models to include full-thickness burn injuries. In our burn time optimization study, reported previously, we identified that the time required to achieve a uniform full-thickness burn in Sprague-Dawley rats was 6 s at 99 °C. To develop biofilm infections in the partial-thickness burn model, we inoculated the wounds with three different levels of *P. aeruginosa*, namely 1 × 10^3^, 1 × 10^4^, 1 × 10^5^ CFU/wound. Given the concerns on animal survival with large (1 × 10^6^ CFU/wound) *P. aeruginosa* inoculums in full-thickness burn injuries shown in earlier studies^11,12,32^, we limited the inoculum to only 1 × 10^3^ and 1 × 10^4^ CFU/wound. The reduced pathogen numbers avoided the lethality associated with greater bacterial inoculums as documented in previous literature^11–13,32^. We also included additional administration of proactive pain relief (Buprenorphine SR LAB) and lactated Ringers resuscitation to all animals remaining in the study beyond PODs 3 and 7. These additional treatments were provided to every animal in those groups in order to maintain uniformity between the different study groups.

Similar to partial-thickness burn wounds, we observed robust infections develop in all the full-thickness scald burns, plateauing above 1 × 10^8^ CFU/g of wound tissue. The wounds inoculated with sterile PBS showed the growth of the native microflora in the burn eschar without any culturable *P. aeruginosa* over the 11 days of the study. Compared to partial-thickness burn wounds infected with *P. aeruginosa*, less tissue separation and sloughing was observed in the full-thickness burn wounds on PODs 7 and 11. However, the full-thickness burns infected with *P. aeruginosa* showed a greater degree of tissue necrosis as seen in the gross wound images in Fig. 1. Interestingly, the wounds inoculated with *P. aeruginosa* showed greater variability in recoverable CFU’s of *P. aeruginosa* compared to our previous work with partial-thickness burns. In the second experimental run, the bacterium was notably absent on both *P. aeruginosa* isolation and standard blood agar plates of the 1 × 10^4^ CFU inoculum on POD 1 and the 1 × 10^3^ CFU inoculum on PODs 3, 7, and 11. This was also confirmed by qPCR for the bacterial DNA indicating that this was not a plating error. However, the bacterium was observed in histological sections of those samples as well as at low levels in end point PCR for the *oprL* gene from different biopsy punches from the same burn wound. Presumably, in that experimental run the native flora may have suppressed the growth or recovery of *P. aeruginosa*, because the first and third runs showed greater recovery of the pathogen from the biopsy punch samples of the 1 × 10^3^ CFU/wound inoculum group. Therefore, sampling errors could be a contributing factor to the recovery of the pathogen in that study group. Conversely, the 1 × 10^4^ CFU/wound inoculum showed better consistency between all three runs, suggesting an optimal infectious dose of *P. aeruginosa* to surface infect full-thickness burn wounds without the mortality observed in previous studies^11,12^. This inoculation method is important because it did not require injection of the pathogen under the burn eschar to establish the biofilm infection, which is commonly used in many murine and rodent burn infection studies^6,7,9,10^. Additionally, the inoculum was spread over the burn surface as planktonic cells and did not require the use of a biomaterial^10^. This model recapitulated the invasive *P. aeruginosa* burn wound infection using a much lower inoculum of the pathogen than previously used, potentially mimicking a more clinically relevant burn infection model than others described^11,12^.

We utilized a new assay that directly measures mRNA to determine expressional changes of important biofilm and virulence associated genes of *P. aeruginosa* in the context of full-thickness burn wound infection. The QuantiGene technology selectively binds mRNA and amplifies the signal of the capture beads specific for the gene of interest. This method avoids the typical challenges associated with reverse transcription of mRNA into cDNA used in qRT-PCR, but instead directly binds to the target mRNA produced by the bacterial cells. The results were expressed as fold change over the planktonic inoculum. The genes involved in alginate biosynthesis (*algD* and *alg8*) and export (*algE*) increased throughout the infection. This indicated that *P. aeruginosa* produced and exported the exopolysaccharide alginate, an important constituent of its mature biofilm matrix. Similar increases occurred in genes associated with biofilm matrix component pel (*pelB*, *pelC*, and *pelD*). However, little to no increase in psl exopolysaccharide (*pslA* and *pslC*) production occurred in the infection. This may be due to the competence this clinical *P. aeruginosa* strain has for producing psl. Similar differences in pel and psl expression have been reported in laboratory *P. aeruginosa* strains PAO1 and PA14^24,33–36^. These two *P. aeruginosa* strains generally express different biofilm matrix components, namely psl (PAO1) and pel (PA14). The strain used in this study may possibly be more pel dominant or potentially deficient for producing psl. We previously characterized the the genome sequence of this clinically isolated pathogen^37^, but work is currently ongoing to further understand the *in vitro* characteristics of this pathogen namely its biofilm formation, motility, and antibiotic resistance genes compared to several other clinical and laboratory isolates of *P. aeruginosa* (manuscript in preparation). Lastly, expression of three virulence genes (*lasA*, *lasB*, and *pvdS*) all showed increases upon *in vivo* infection, indicating adaptation to the wound environment by increasing production of siderophores to liberate iron from host tissues and elastase to degrade the host tissue matrix^38–41^. Scavenging bound iron from the blood and tissues within the burn wound is an important step for *P. aeruginosa* biofilm formation^42,43^. Taken together the increased expression of biofilm exopolysaccharides along with factors that degrade host tissues and liberate important nutrients indicates active biofilm development by *P. aeruginosa* in full-thickness burn wounds.

Initial characterization of the host response to the tissue damage resulting from the full-thickness burn and subsequent *P. aeruginosa* infection suggests marked differences from partial-thickness burns. Our work in deep partial-thickness burns showed both a local and systemic, inoculum-dependent, neutrophil response to the *P. aeruginosa* infection. Interestingly, in the full-thickness burn, a similar elevation of neutrophil activity with *P. aeruginosa* infection, regardless of inoculum level, above the burn trauma itself was notably absent. A general trend of increased MPO activity was observed as time progressed in response to the burn, but not necessarily to the presence of *P. aeruginosa* in the burn eschar. Additionally, only on POD 11 was an increase in systemic neutrophil counts seen in only the 1 × 10^4^ CFU/wound inoculum group as compared to all the other inoculation groups. The increased neutrophil counts were accompanied with a large standard deviation, because the two samples from the final experimental run of the 1 × 10^4^ CFU/wound inoculum clotted before they could be analyzed, while the first run showed lower counts (~10 × 10^3^ neutrophils/µl) and the second run had higher counts (~34 × 10^3^ neutrophils/µl). Comparatively, in the partial-thickness burn with *P. aeruginosa* infection, we previously reported a trend of increased systemic neutrophils by POD 7 and significant increases on POD 11^22^. The lack of neutrophil response to the subsequent infection of full-thickness burns with *P. aeruginosa* may be due to the lack of blood flow or circulation in the burn eschar. In the deep partial-thickness burn model, roughly the bottom third of the dermis remained vitalized, while the full-thickness burn dermis was devoid of blood circulation. In larger TBSA rat burn models, slow accumulation of neutrophils into full-thickness burned skin, due to limited number of blood vessel beneath the burned dermis, has previously been shown^44^. Similarly, sequestration of neutrophils into distal injury sites (polyurethane sponges or bacterial skin lesions) from the burn was reduced with concomitant presence of full-thickness burn injuries^45,46^. These intrinsic features of the burn wound, albiet in larger TBSA burns, may have reduced the host’s ability to interact with *P. aeruginosa* or native microflora in the burn injury, thereby shielding the invading microbes from the innate immune response. Further, in-depth analysis (local and systemic cytokine expression, histologic scoring of infected verus un-inoculated tissue sections, circulating damage/pathogen associated molecular patterns, and systemic immune cell counts) of the host response to both partial- and full-thickness burns in the context of *P. aeruginosa* biofilm burn wound infection will be reported in a future manuscript.

In this report, we detail the course of *P. aeruginosa* infection in a 10% TBSA full-thickness scald burn with Sprague-Daweley rats. Robust infections developed using relatively low inoculums of 1 × 10^4^ *P. aeruginosa* cells spread over the surface of the burn wound instead of injecting the inoculum beneath the burn eschar. The bacterial infection resulted in the natural development of *P. aeruginosa* biofilms in the burn eschar, coupled with systemic infection by 11 days post burn and inoculation. This work further enhances our portfolio of rat scald burn biofilm infection models for testing the effectiveness of novel and easily deployable anti-biofilm treatments in the context of both severe partial- and full-thickness burn wounds that afflict combat soldiers on the battlefield.

## Materials and Methods

### Animal ethics statement

Research was conducted in compliance with the Animal Welfare Act, the implementing Animal Welfare Regulations, and the principles of the Guide for the Care and Use of Laboratory Animals, National Research Council. The United States Army Institute of Surgical Research (USAISR) Institutional Animal Care and Use Committee approved all research conducted in this study (Animal Protocol A-16–047) on 15 September 2016. The facility where this research was conducted is fully accredited by AAALAC International.

### Full-thickness burn and infection

Full-thickness burns were made on the dorsum of 61 male Sprague-Dawley rats using the method we previously described^22^. The burn area averaged ~10% of the total body surface area based on a body weight range between 350–450 g in Meeh’s formula, $A=k{W}^{\frac{2}{3}}$, where A is total body surface area (cm^2^), *k* is Meeh’s constant (9.46), and *W* is body weight (g)^47^. In brief, the animals were acclimated to the facility for 2 weeks prior to the burn. The day before the burn, the dorsal surface of anesthetized (Forane, Baxter Healthcare Corporation, Deerfield, IL) rats was shaved and depilated (Nair, Church & Dwight Co, Ewing, NJ). Additionally, 1.2 mg/kg of Buprenorphine SR LAB (Zoopharm Pharmacy) was subcutaneously administered for proactive pain management. Post-shaving, the rats were housed individually until the end of the experiment.

Immediately prior to the burn, rats were anesthetized with 2.5% isoflurane (Forane) for 15 minutes in an induction chamber followed by application of eye lube (Artificial Tears Ointment, Akorn, Inc., Lake Forrest, IL). Pulse rate and blood oxygen levels (2500A Vet Pulse Oximeter, Nonin Medical, Inc., Plymouth, MN) were monitored throughout the procedure. The anesthetized rats were positioned into a custom burn template, which was lowered into 99 °C water bath for 6 seconds. We previously showed that this exposure time produced full-thickness burns in Sprague-Dawley rats^22^. Immediately post-burn, the burn area was blotted on damp (room temperature) paper towels to remove excess water and the first of four fluid resuscitations (warm 4 ml Lactated Ringers Solution (LRS) based on Parkland’s Formula) given via intraperitoneal injection. The remaining resuscitations were given within 36 hours following the burn. Burn wounds were imaged using a Nikon D90 with Nikkor AF-S lens (18–105 mm, A:3.5–5.6 G) following the burn and on the day of euthanasia.

Immediately after the burn and first resuscitation, *P. aeruginosa* strain 12-4-4(59) suspended in 1 × phosphate-buffered saline (PBS) was inoculated on the burn surface at 1 × 10^3^ or 1 × 10^4^ CFU/wound^11,37^. The bacterial cells were grown overnight at 37 °C in Trypticase Soy Broth (TSB; Becton, Dickerson and Company, Sparks, MD) and subcultured the day of the burn in fresh TSB to mid-logarithm growth phase. Prior to inoculation, the bacterial cells were centrifuged at 3,000 × g for 15 minutes at 4 °C and re-suspended in PBS to the correct cell density. A 100 µl *P. aeruginosa* suspension was spread over the burn area with a sterile pipette tip. Un-inoculated control wounds received 100 µl of sterile PBS. In total 61 animals comprised the study, with 12 serving as un-inoculated controls and 24 in each *P. aeruginosa* inoculum group. The study was repeated three times. Each experimental repeat consisted of one third of the animals in each group, distributed between four end points, post-operative day (POD) 1, 3, 7, and 11. One animal in the 1 × 10^4^ CFU/wound inoculum required a replacement due to early removal from the study on POD 5.

To prevent disruption of the burn surface by the animals, the wounds were sealed with Tegaderm^TM^ Film (3M Health Care, St. Paul, MN) using NOTAPE professional silicone bonding adhesive (Vapon, Inc. Fairfield, NJ). N-terface Wound Contact Layer (Winfield Laboratories, Inc. Richardson, TX) separated the burn surface and the Tegaderm^TM^ Film. Rats were additionally placed into composite rat jackets, described previously for the duration of the study^22^. A ThermoCare Portable Animal Intensive Care Unit (Daisy Products LLC, Paso Robles, CA) set to 37 °C was used to recover the animals after the burn until the effects of the anesthesia wore off. Additional fluid resuscitation (LRS given *intraperitoneally*) and pain relief (Buprenorphine SR LAB given *subcutaneously*) beyond the initial 36 hours following the burn were provided on PODs 3 and 7 to rats remaining in the study beyond those time points. Daily evaluation of body weight and symptoms of pain/distress were made throughout the study.

### End point procedures

For euthanasia, rats were anesthetized with 100 mg/kg Ketamine HCl (Zetamine, MWI Veterinary Supply Co. Boise, ID) and 10 mg/kg Xylazine (Akron Animal Health, Inc. Lake Forrest, IL) administered *intraperitoneally*. Isoflurane (4%) was given as needed during the terminal procedure. A cardiac puncture was used to obtain a blood sample for systemic neutrophil counts (EDTA Vacutainer 367841, Becton, Dickerson and Company, Sparks, MD) using an Abbott CELL-DYN^®^ 3700 Blood Count Analyzer (Abbott Laboratories, Abbott Park, IL). Additionally, in the second and third runs of the study, 2.0 ml of blood was aseptically aliquoted into Oxoid^TM^ Signal^TM^ Blood Culture System bottles (Thermo Fisher Scientific, Waltham, MA) to detect the presence of bacterial cells in the systemic circulation. Bacterial cells found in the systemic circulation were identified using a GEN III OmniLog® Combo System (Biolog, Inc. Hayward, CA). Fatal-Plus^®^ (Vortech Pharmaceuticals, LTD. Dearborn, MI) was administered intra-cardiac and euthanasia confirmed by lack of cardiac movement, pulse, and breathing as defined by the AVMA Guidelines for the Euthanasia of Animals: 2013 Edition^48^.

Upon removal of the Tegaderm and N-terface dressings, the wounds were imaged and entire burn area excised from the dorsum. Three (cranial, middle, and caudal) separate histologic cross-sections of the burn area were fixed in 10% buffered formalin in PBS (Fisher Diagnostics, Kalamazoo, MI) for 48 hours and processed for routine embedding in paraffin wax. Biopsy punch (7 mm) samples of the burn area were isolated for quantitative bacteriology, scanning electron microscopy, myeloperoxidase (MPO) activity, and bacterial mRNA analysis. Internal organs (spleen, liver, kidney, and lungs) were also recovered. Tissue and organ samples, except those for quantitative bacteriology, were flash frozen in liquid Nitrogen and kept at −80 °C prior to analysis.

### Quantification of bacterial load

The samples (biopsy punches) used to determine the bacterial load within the burn tissue were homogenized in 1.0 ml of PBS in MagNA Lyser Green Beads tubes (Roche Diagnostics GmbH, Mannheim, Germany) using a FastPrep®-24 Tissue Homogenizer (MP Biomedicals, LLC. Santa Ana, CA). The homogenized samples were plated in duplicate on Trypticase soy agar containing 5% sheep’s blood (Becton, Dickinson and Co. Sparks MD) and *P. aeruginosa* isolation agar (Hardy Diagnostics, Santa Maria, CA). Viable bacterial colonies were counted and plotted as log_10_ (CFU/g wound tissue) ± Standard Deviation.

Bacterial DNA was also isolated from the same biopsy punch samples for viable CFU quantification using PCR as previously described^22^. Fifty microliters from each of the four-biopsy sample tissue homogenates per animal were pooled and DNA isolated using the DNeasy Blood & Tissue Kit (Qiagen, Valencia, CA). To quantify bacterial load in the burn eschar, primers and probes for outer membrane lipoprotein *oprL* of *P. aeruginosa*^49^ and universal primers and probes for the 16S rDNA of gram-negative bacterial cells were used^50^. Primer and probe sequences are listed in Supplemental Table 1. Standard curves were generated from mid-log growth phase cultures of *P. aeruginosa*. Genomic DNA concentration was measured using a Quant-iT ds DNA BR Assay Kit (Invitrogen, Carlsbad, CA) and confirmed using gel separation. All samples were run in triplicate on a StepOne Plus Real-Time PCR System (Applied Biosystems, Inc. Foster City, CA) using a total volume of 20 µl of TaqMan Gene Expression Master Mix (Applied Biosystems). Concentrations of forward and reverse primers and TaqMan MGB probe were set to 100 nM along with 5 µl of sample template. Target DNA was amplified using a standard qPCR protocol of a 5 minute 95 °C denaturation step, followed by 40 PCR amplification cycles (95 °C for 15 s and 60 °C for 60 s). The resulting data was analyzed using the StepOne software provided with the instrument. The genome copy number was calculated from the amount of genomic (*P. aeruginosa* or total) DNA and normalized by the weight of the tissue to provide CFU/g tissue.

### Histology

Thin 5 µm sections of fixed rat burn eschar were stained with hematoxylin-eosin (H&E) or H&E with Giemsa to evaluate tissue damage and *P. aeruginosa* invasion into the tissue. Images were taken with both the 20× and 63× objectives of a Leica Aperio Versa 200 slide scanner (Leica Biosystems, Inc. Buffalo Grove, IL). Additionally, peptide nucleic acid (PNA)-fluorescent *in situ* hybridization (FISH) was used to confirm the presence of *P. aeruginosa* deep within the burned tissue section using a standardized method in our laboratory^51^. The *E. coli*/*P. aeruginosa* PNA FISH^®^ Kit was used to visualize the penetration of *P. aeruginosa* into the burn eschar. Briefly, tissue sections were incubated with AvanDx fixative solution for 20 minutes at 55 °C. The PNA-FISH probe was incubated with each tissue section for 45 minutes allowing hybridization of the probe to the bacterial cells. The tissue sections were then washed with wash buffer, supplied with the kit, for 45 minutes. Tissue sections were mounted with the supplied mounting medium and edges of the coverslip sealed with nail polish. Specimens were imaged using the Texas Red filter (596/615 nm) and DAPI filter (358/461) with both 20× and 63× objectives of the Leica Aperio Versa 200 slide scanner.

### Scanning electron microscopy

Both the surface and tissue cross-section of the burn eschar were evaluated by scanning electron microscopy (SEM). To visualize the surface of the wound, seven millimeter biopsy punch samples were fixed with 2.5% phosphate-buffered glutaraldehyde for at least 24 hours at 4 °C. Fixed specimens were processed through a graded series dehydration using cold ethanol/water (10%, 30%, 50%, 70%, 80%, 90%, 95%, 100%) and an incubation time of 10 minutes followed by critical point drying (EM CPD300, Leica Biosystems Inc. Buffalo Grove, IL). Immediately prior to examination, the wound biopsy punch samples were coated with carbon and gold/palladium (Leica ACE600 Coater). The histological cross-sections were deparaffinized with 100% xylene and air dried prior to coating with carbon and gold/palladium. Both sets of specimens (biopsy punches and tissue cross-sections) were visualized using a Sigma VP40 field emission scanning electron microscope (Carl Zeiss, Inc. Germany) in high vacuum mode at 2 kV.

### QuantiGene plex gene expression assay for key *P. aeruginosa* biofilm and virulence genes

We used the QuantiGene Plex Assay (Assay ID M17102001, Affymetrix, Inc. Santa Clara, CA) to determine the expression level of key biofilm structural genes (*algD*, *alg8*, *algE*, *pelB*, *pelC*, *pelD*, *pslA*, and *pslC*) and virulence genes (*lasA*, *lasB*, and *pvdS*) using three distinct housekeeping genes (*proC*, *fabD*, and *rpoD*) following the manufacturer’s procedure for Frozen Tissue Homogenates. Briefly, samples were pulverized under liquid Nitrogen using a Bessman Tissue Pulverizer (Spectrum, Inc. Rancho Dominguez, CA) and lysed for 15 minutes at 65 °C with lysis mixture (cold TES Buffer with Lysozyme and Proteinase K Homogenization solution). The supernatants of the tissue homogenates were stored at −80 °C, after centrifuging at 4,000 rpm for 5 minutes, until analysis. The tissue homogenates were thawed and incubated at 37 °C for 30 minutes prior to mixing with the Working Bead Mix, containing Proteinase K and the Capture Beads, in a hybridization plate and incubated at 54 °C for 18 hours with gentle shaking at 600 rpm. After the overnight incubation, the sample was transferred to the Magnetic Separation Plate. Using the Hand-held Magnetic Plate Washer, the samples were washed 3× with Wash Buffer in between 1 hour incubations at 50 °C with Pre-Amplifier Solution, Amplifier Solution, and Label Probe Solution. After the Label Probe Solution, SAPE Working Reagent was added for 30 minutes at room temperature. After washing the plate 3× with SAPE Wash Buffer, 130 µl of SAPE Wash Buffer was added to each sample and mixed for 3 minutes at 800 rpm followed by immediate reading on a Bio-Plex 200 System with Bio-Plex Manager^TM^ Software Version 6.1 Build 727 (BioRAD Laboratories, Inc. Hercules, CA).

### Quantification of myeloperoxidase activity in burn tissue

A Bessman Tissue Pulverizer was used to fracture biopsy punch samples under liquid Nitrogen. The samples were further homogenized as directed by the Fluoro MPO Myeloperoxidase Detection Kit (Cell Technology, Inc. Mountain View, CA). Briefly, an IKA T10 basic Ultra Turrax tissue homogenizer (IKA Works, Inc. Wilmington, NC) was used to process the samples in MPO homogenization buffer, provided in the kit. The samples were then centrifuged at 12,000 × g for 20 minutes at 4 °C and re-suspended in MPO solubilization buffer containing 0.5% Hexadecyltrimethylammonium (Sigma-Aldrich, Inc. St. Louis, MO). The re-suspended samples were homogenized again and sonicated using a Sonic Dismembrator Model 100 (Fisher Scientific, Kalamazoo, MI) for 30 s followed by two cycles of freezing/thawing before isolating the supernatant. Equal volumes of sample or MPO enzyme standard were mixed with MPO reaction cocktail and incubated in black opaque, clear bottom, microtiter plates for 30 minutes at room temperature. Fluorescence (530/590 nm) was measured using a BioTek Synergy HT with Gen5 Software (BioTek Instruments, Inc. Winooski, VT). MPO activity of each sample was calculated from the MPO enzyme standard curve and plotted as MPO activity in milliUnits (mU) ± Standard Deviaiton.

### Statistical analysis

All data were plotted and analyzed using GraphPad Prism 7.03 (GraphPad Software, Inc. San Diego, CA). Comparisons were performed using two-way ANOVA with Tukey’s correction for multiple comparison and an α set to 0.05. Data was graphed as the mean ± Standard Deviation (SD).

### Disclaimer

The opinions or assertions contained herein are the private views of the author and not to be construed as official or as reflecting the views of the Department of the Army or the Department of Defense.

## Supplementary information

Supplementary Information

## Data Availability

The datasets generated during and/or analysed during the current study are available from the corresponding author on reasonable request.
