# Supplementary material for: Formation of Pseudomonas aeruginosa Biofilms in Full-thickness Scald Burn Wounds in Rats
Source: Sci Rep. 2019 Sep 20;9:13627. doi: 10.1038/s41598-019-50003-8 (PMC6754504; doi:10.1038/s41598-019-50003-8)
Supplement: Supplementary file 1 — Supplementary Information [file 41598_2019_50003_MOESM1_ESM.pdf]

Formation of *Pseudomonas aeruginosa* Biofilms in Full-thickness Scald Burn Wounds in Rats

Kenneth S. Brandenburg, PhD; Alan J. Weaver Jr., PhD; S. L. Rajasekhar Karna, PhD; Tao You, PhD; Ping Chen, PhD; Shaina Van Stryk, BS; Liwu Qian, MD/PhD; Uzziel Pineda; Johnathan J. Abercrombie, MS; and Kai P. Leung*, PhD

Supplementary Information

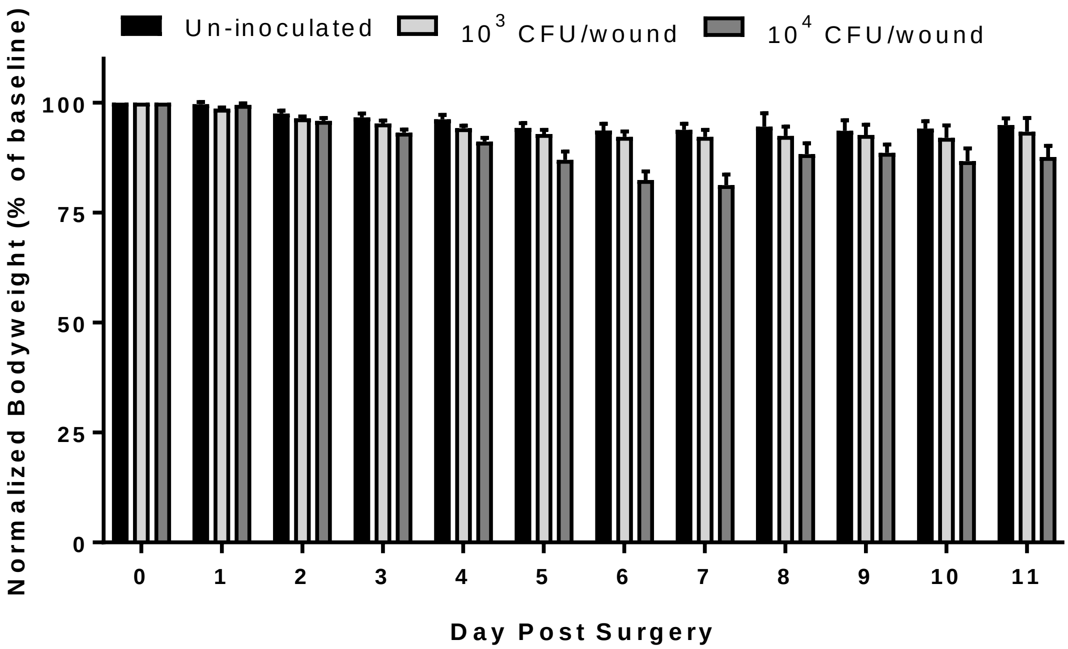

Supplementary Figure 1

Supplementary Figure 1: Normalized Body Weights over 11 days post burn and inoculation with sterile PBS, 1×10^3 CFU/wound *P. aeruginosa*, or 1×10^4 CFU/wound *P. aeruginosa*.

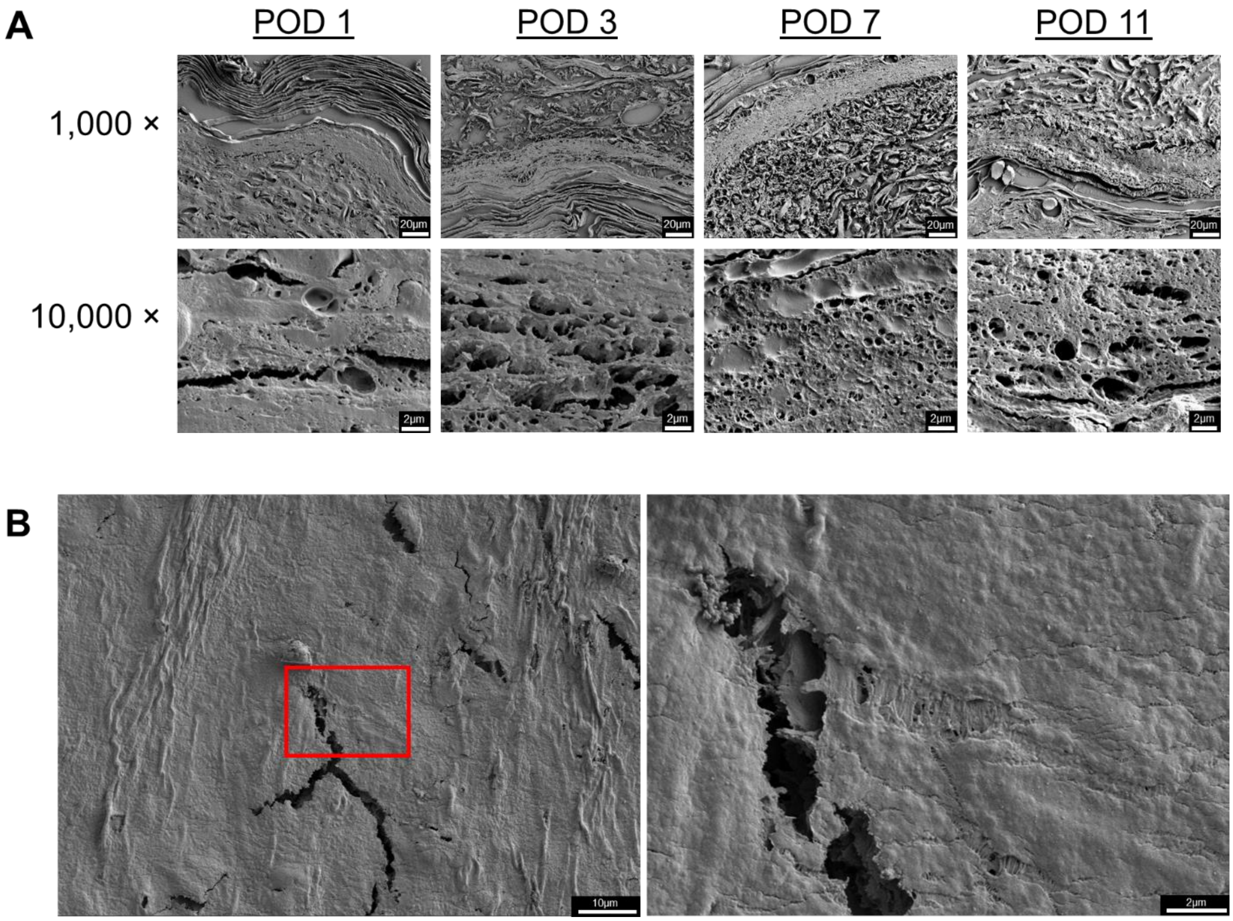

Supplementary Figure 2

Supplementary Figure 2: Representative scanning electron micrographs of control burn wounds inoculated with PBS. The lack of *P. aeruginosa* is notable in the histologic cross-sections (A) of the burn wound over the 11 day course of the experiment (Scale bar of 1,000× images is 20µm and 10,000× images is 2µm) and on the burn surface (B) on POD 11 (Scale bar of 1,000× image is 10µm and 10,000× image is 2µm).

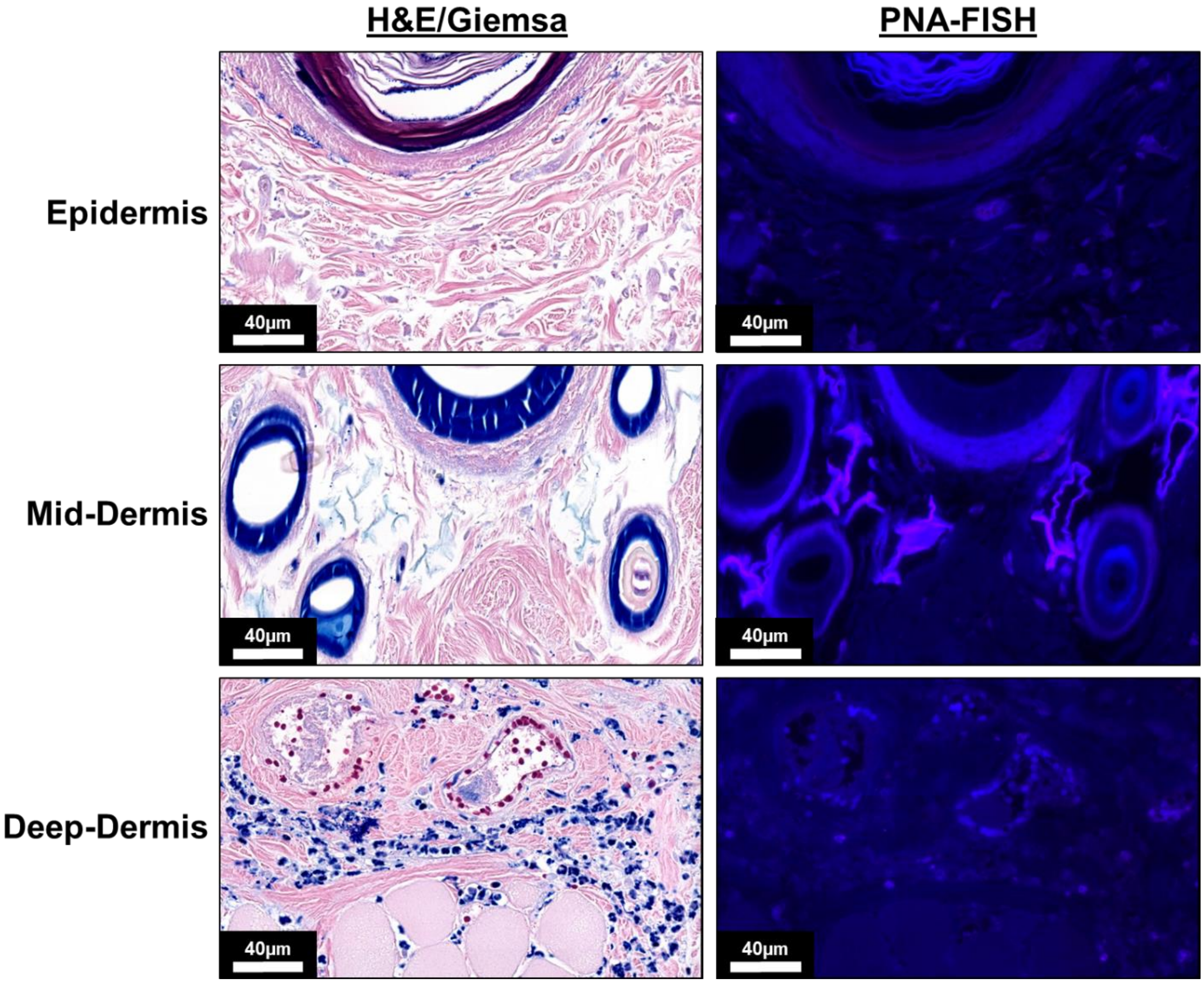

Supplementary Figure 3

Supplementary Figure 3: Representative micrographs of the control burn wound tissue inoculated with PBS on POD 7 stained with H&E with Giemsa or PNA-FISH with primers specific for *P. aeruginosa*. Images show three levels of the burned skin including the epidermis, middle of the dermis, and the deep dermis near the panniculus carnosus. Bacterial cells, mostly gram-positive cocci, were observed in the burn wounds inoculated with PBS, but *P. aeruginosa* was not observed in either the H&E with Giemsa or PNA-FISH stained sections. Scale bars are 40µm.

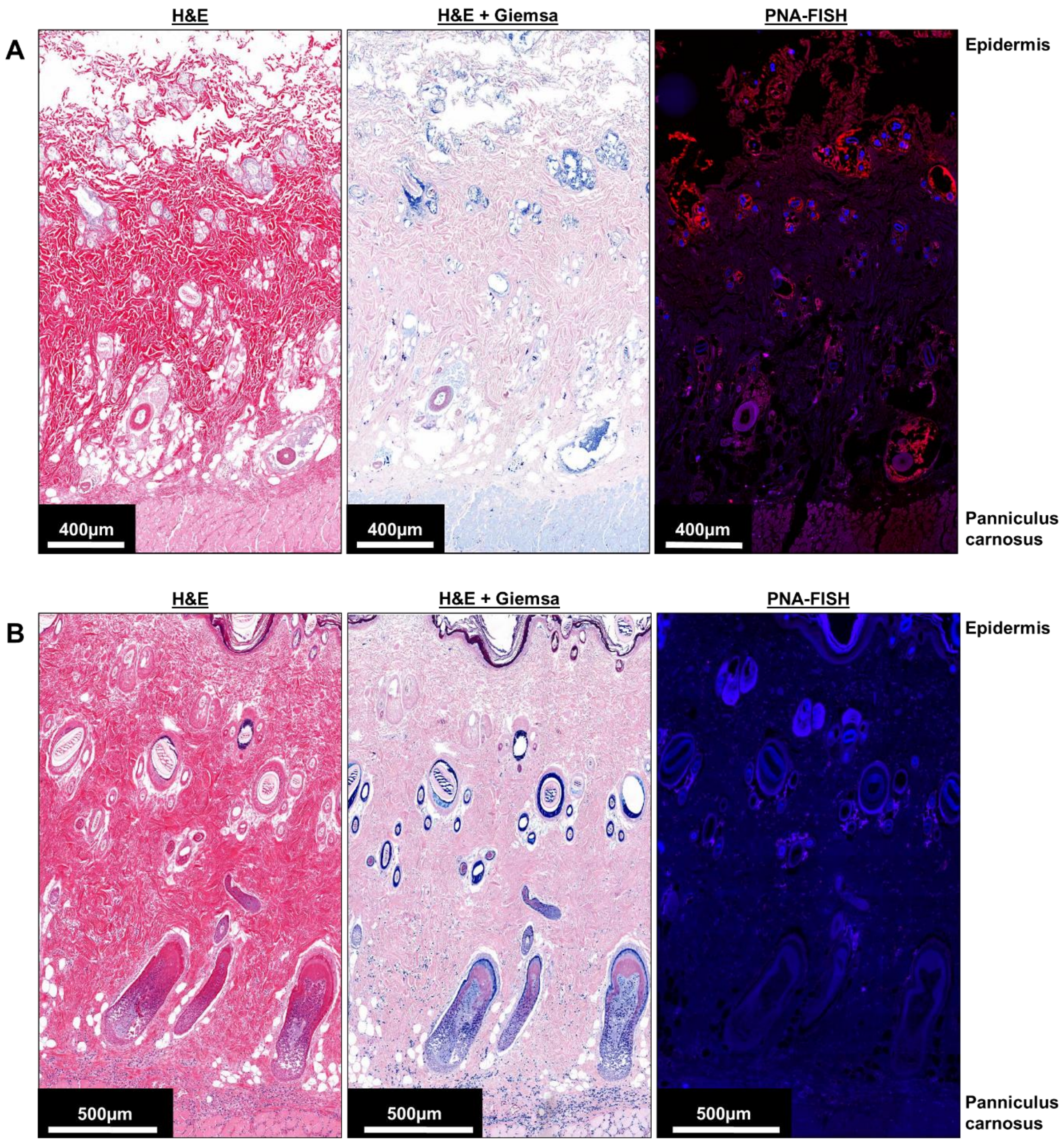

Supplementary Figure 4

Supplementary Figure 4: Overview of POD 7 tissue cross-sections (Epidermis to Panniculus carnosus) inoculated with 1×10^4 CFU/wound of *P. aeruginosa* (A) or PBS (B) stained with H&E, H&E with Giemsa, or PNA-FISH with primers specific for *P. aeruginosa*. Scale bars are A) 400µm and B) 500µm.

Supplementary Table 1. List of PCR primers for 16s ribosomal target regions and qRT-PCR	
Gene Name	Primer Sequence 5’ to 3’
P. aeruginosa oprL – forward	5’-ATGGAAATGCTGAAATTCGGC-3’
P. aeruginosa oprL – reverse	5’-CTTCTTCAGCTCGACGCGACG-3’
P. aeruginosa oprL – probe	(6FAM)-5’-TGCGATCACCACCTTCTACTTCGAGT-3’-MGBNFQ
Total 16S rDNA – forward	5’-TCCTACGGGAGGCAGCAGT-3’
Total 16S rDNA – reverse	5’-GGACTACCAGGGTATCTAATCCTGTT-3’
Total 16S rDNA – probe	(6FAM)-5’-CGTATTACCGCGGCTGCTGGCAC-3’-MGBNFQ
